# Ecological speciation in the tropics: insights from comparative genetic studies in Amazonia

**DOI:** 10.3389/fgene.2014.00477

**Published:** 2015-01-21

**Authors:** Luciano B. Beheregaray, Georgina M. Cooke, Ning L. Chao, Erin L. Landguth

**Affiliations:** ^1^Molecular Ecology Lab, School of Biological Sciences, Flinders UniversityAdelaide, SA, Australia; ^2^The Australian Museum, The Australian Museum Research InstituteSydney, NSW, Australia; ^3^Departamento de Ciências Pesqueiras, Universidade Federal do AmazonasManaus, Brazil; ^4^National Museum of Marine Biology and AquariumPintung, Taiwan; ^5^Division of Biological Sciences, University of MontanaMissoula, MT, USA

**Keywords:** biogeography, adaptive divergence, evolutionary landscape genetics, phylogenetics, phylogeography, ecological genomics, tropical diversification, biodiversity conservation

## Abstract

Evolution creates and sustains biodiversity via adaptive changes in ecologically relevant traits. Ecologically mediated selection contributes to genetic divergence both in the presence or absence of geographic isolation between populations, and is considered an important driver of speciation. Indeed, the genetics of ecological speciation is becoming increasingly studied across a variety of taxa and environments. In this paper we review the literature of ecological speciation in the tropics. We report on low research productivity in tropical ecosystems and discuss reasons accounting for the rarity of studies. We argue for research programs that simultaneously address biogeographical and taxonomic questions in the tropics, while effectively assessing relationships between reproductive isolation and ecological divergence. To contribute toward this goal, we propose a new framework for ecological speciation that integrates information from phylogenetics, phylogeography, population genomics, and simulations in evolutionary landscape genetics (ELG). We introduce components of the framework, describe ELG simulations (a largely unexplored approach in ecological speciation), and discuss design and experimental feasibility within the context of tropical research. We then use published genetic datasets from populations of five codistributed Amazonian fish species to assess the performance of the framework in studies of tropical speciation. We suggest that these approaches can assist in distinguishing the relative contribution of natural selection from biogeographic history in the origin of biodiversity, even in complex ecosystems such as Amazonia. We also discuss on how to assess ecological speciation using ELG simulations that include selection. These integrative frameworks have considerable potential to enhance conservation management in biodiversity rich ecosystems and to complement historical biogeographic and evolutionary studies of tropical biotas.

## INTRODUCTION

“Natural selection, as we shall hereafter see, is a power incessantly ready for action …”

(Darwin, 1859)

Evolution creates and sustains biodiversity via adaptive changes in ecologically relevant traits. Understanding how organisms adapt and diversify have been topics of fundamental importance in biology for over 150 years (Losos et al., 2013). It is now realized that evolution reflects both historical and contemporary contingencies and is mediated by bidirectional interactions between ecological and evolutionary changes (Schoener, 2011; Losos et al., 2013). Thus, to understand adaptive changes and patterns of biological diversification it is often necessary to integrate information from both past (e.g., geomorphologic and paleoclimatic variation) and recent (e.g., natural selection) processes (Avise, 2000; Hoffmann and Sgro, 2011; Losos et al., 2013). Bridging macro and microevolutionary studies provides ways to clarify regional patterns of biological diversification and their underlying mechanisms of speciation. Here we focus on the contribution of studies that simultaneously assesses historical and ecologically driven signatures of population divergence to explain patterns of biodiversity in tropical ecosystems.

Speciation – the continuous process that gives rise to biological diversity, is often intimately associated to changes in phenotypes and to adaptation to the ecological environment (Darwin, 1859; Mayr, 1963; Dobzhansky, 1970). Natural selection contributes to ecological adaptation both in the presence or absence of geographic isolation between populations, and as a consequence, drives phenotypic diversification, population divergence, the evolution of reproductive isolation and the formation of new species (Endler, 1977, 1986; Schluter and Conte, 2009; Nosil, 2012). Ecologically mediated selection is considered an important driver of speciation (Schluter and Nagel, 1995; Schluter, 2001; Shafer and Wolf, 2013). Yet, ecological mechanisms driving reproductive isolation vary in space and time and can have different phenotypic and genetic signatures (Nosil, 2012), making it difficult to assess the role and the proportional contribution of natural selection in the origin of species. On the other hand, non-mechanistic spatial frameworks are generally easier to establish and to test, and as such have historically dominated the study of speciation. Such frameworks tend to focus on the geographic arrangement of populations undergoing reproductive isolation (e.g., allopatric, parapatric, or sympatric) instead of the processes driving the evolution of reproductive isolation. These studies have shown that allopatric speciation is common across taxonomic groups and biomes (Mayr, 1942; Coyne and Orr, 2004). They have also promoted the view that speciation primarily results from genetic drift due to geographic isolation over that of speciation via natural selection (Schluter, 2001; Via, 2001; Rundle and Nosil, 2005).

The increasing integration between molecular genetic approaches with theoretical and empirical ecological studies (both in the field and experimentally; Nosil, 2012; Shafer and Wolf, 2013) seen during the last 20 years has restored excitement about the role of ecology in speciation, returning to Darwin’s (1859) original theory that divergence may be driven by adaptation rather than merely being a by-product of geographic isolation. The term ‘ecological speciation’ was coined during this period (Schluter, 1996) and can be defined as “the process by which barriers to gene flow evolve between populations as a result of ecologically based divergent selection between environments” (Nosil, 2012). During ecological speciation, populations occupying different biotic or abiotic environments or using different resources experience unique selection pressures that directly or indirectly result in reproductive isolation, even if the original populations remain in contact (Schluter, 2001). The historical foundations of ecological speciation are inextricably linked to the works of Darwin, Mayr, and Dobzhansky, which provide the reference with which 21st century views of speciation should be contrasted (Harrison, 2012). As is often the case in evolutionary biology research, ecological speciation has not been free of criticism. These include discussions about the value of this terminology, its equivalency to parapatric and sympatric speciation, and the role of divergent natural selection in the speciation process (readers interested in contrasting views to those presented here should consult Sobel et al., 2010 and Harrison, 2012).

Ecological speciation can occur under several spatial scenarios – from allopatry through to sympatry (Schluter, 2001; Coyne and Orr, 2004; Rundle and Nosil, 2005), with classical examples being parapatric (Endler, 1977; Sobel et al., 2010). Ecological divergence and reproductive isolation can evolve under a single or under multiple geographic scenarios (e.g., it could begin in allopatric and be completed in sympatric conditions, Rundle and Schluter, 2004). The geographic arrangement of populations is important because it affects the source of selection and rates of gene flow, but what is essential in ecological speciation is that the divergence be primarily driven by divergent selection (Nosil, 2012). Indeed, substantial population divergence and speciation is known to occur between ecologically dissimilar populations even in the face of ongoing gene flow between these (i.e., divergence-with-gene-flow; Smith et al., 1997; Beheregaray and Sunnucks, 2001; Schluter, 2001; Coyne and Orr, 2004; Schluter and Conte, 2009; Bernatchez et al., 2010; Cooke et al., 2012a; Sexton et al., 2014). Identifying these incipient ecological species represents an opportunity to investigate ongoing evolutionary processes in situations where adaptive divergence and reproductive isolation are associated (for a review see Orr and Smith, 1998). Allopatric ecological speciation might also be common in situations where ecological divergence is a stronger driver of reproductive isolation than genetic drift (Funk, 1998; Vines and Schluter, 2006).

**FIGURE 1:**
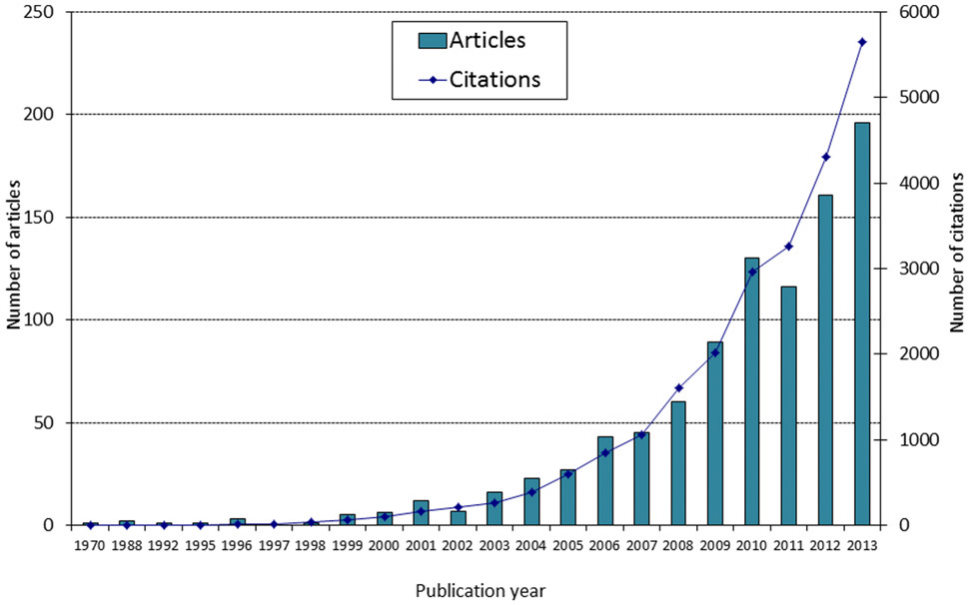
**Number of articles published under the topic “ecological speciation” and corresponding number of citations (source: Web of Science, May 2014)**.

Studying the role of ecology in the speciation process is a popular research area these days. Our Web of Science® search conducted in May 2014 indicated that 996 articles were published under the topic of “ecological speciation,” with 862 of these being empirical studies. Interest in this research topic has grown exponentially in recent years, and so has the corresponding citation rate (**Figure 1**). For instance, 2013 has seen almost 200 publications and over 5,600 citations, compared to 60 articles and 1,600 citations in 2008. Interestingly, our evaluation shows that the majority of empirical studies (674 out of 862) used information from genetic datasets or genetic knowledge to improve pattern interpretation. Indeed, the genetics of ecological speciation is becoming increasingly studied across a variety of taxa and environments (Wolf et al., 2010; Nosil, 2012; Shafer and Wolf, 2013). As a result, speciation research has expanded beyond traditional boundaries, since genetic and genomic techniques now offer the means to disentangle the manifold (albeit complex) processes of drift from selection, across various evolutionary stages, environments and taxa (Moritz et al., 2000; Shafer and Wolf, 2013; Seehausen et al., 2014).

The initial aim of this paper is to examine the literature to assess the research interest about ecological speciation in the tropics, the region that encompasses the most-species rich ecosystems on Earth. Tropical rainforests and coral reefs house a disproportionally high number of species and, as a consequence, have become the focus of great attention of scientists and the general public (Primack, 2014). For instance, even though tropical rainforests occupy only 7% of the Earth’s land area, they are estimated to contain most of the Earth’s species (Corlett and Primack, 2010). Although several opinions, reviews, and a few meta-analyses have been published about speciation in the tropics (e.g., Haffer, 1997; Moritz et al., 2000; Wiens and Donoghue, 2004; Rull, 2008; Hoorn et al., 2010b; Salisbury et al., 2012; Bowen et al., 2013; Smith et al., 2014), to the best of our knowledge none have focused on ecological speciation. To address this shortcoming, we examine the empirical literature to identify analytical approaches that have been used to study ecological speciation in the tropics.

Motivated by a review about diversification of rainforest faunas (Moritz et al., 2000) and by our initial assessment of the literature, we further argue for analytical approaches that explore genomic (or genetic) datasets at the population level and thus bridge historical and ecological considerations in tropical speciation research. We perceive the need for a research program that simultaneously addresses large-scale biogeographical and taxonomic questions in the tropics, while effectively assessing finer-scale relationships between reproductive isolation and ecological divergence.

To contribute toward this goal, we propose a framework for ecological speciation research that integrates information from evolutionary landscape genetics (ELG), genome scans, population genetics, phylogeography, and phylogenetics. We then use published population-level genetic datasets from five codistributed and taxonomically diverse Amazonian fish species (Cooke et al., 2012a,b,c,d, 2014) to illustrate the performance of the framework in studies of tropical speciation. Specifically, these studies examine the accumulation of population and lineage diversity in these fish groups within the context of geomorphological history, tributary arrangement, and divergent natural selection putatively associated with hydrochemical ecotones (i.e., at the interface of major rivers with different ‘water colors’). Although these lineages show different biogeographic and evolutionary histories, striking commonalities exist in the way that population genetic divergence and incipient speciation appear to be ecologically induced and spatially influenced by ecotones.

We suggest that this framework can assist in distinguishing the relative contribution of natural selection from biogeographic history in the origin and maintenance of biodiversity, even in a complex and understudied tropical ecosystem such as Amazonia. In addition, we show that it is feasible to frame ecological speciation in a spatially explicit ELG context that includes selection. We also discuss recent developments in environmental mapping, simulations and population genomics that allow clarification of population divergence across selection gradients.

## WHY IS THE RESEARCH EFFORT IN ECOLOGICAL SPECIATION SO LOW IN TROPICAL ECOSYSTEMS?

Our assessment of empirical research productivity in ecological speciation shows that the tropics have been largely left behind compared to other ecosystems. Only 51 publications (5.2%) out of the 996 previously identified articles (**Figure 1**) dealt with tropical species, and only 40 of those could be classified as empirical studies. Although our searches might have missed relevant studies that did not use the words “tropical” or “tropic(s)” in the abstracts, keywords, or titles, our inspection of the literature indicates that research effort in ecological speciation is indeed largely biased toward temperate regions. This conclusion is consistent with the empirical literature reviewed in a recently published book on ecological speciation (Nosil, 2012), with a review of the topic (Rundle and Nosil, 2005) and with several syntheses about tropical diversification (Moritz et al., 2000; Rull, 2008; Bowen et al., 2013).

**FIGURE 2:**
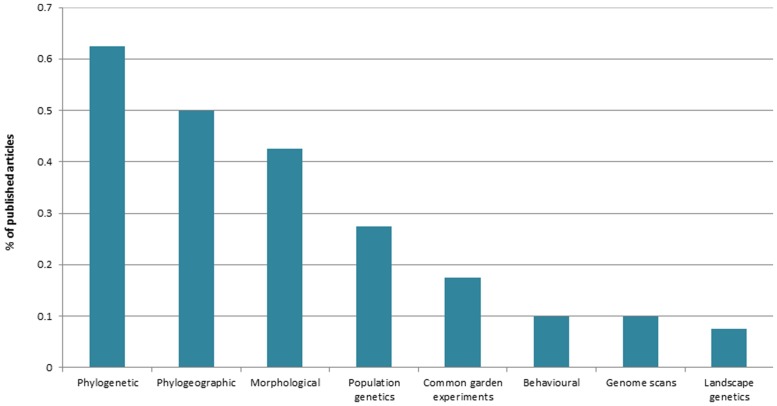
**Main approaches used to study ecological speciation in the tropics (source: Web of Science, May 2014)**.

Research trends in tropical ecological speciation were collected by inspecting the 40 recovered empirical articles for key selected categories. In terms of biological realm, most publications focused on terrestrial (65%), rather than marine (20%), or freshwater (15%) organisms. Terrestrial plants and terrestrial invertebrates were the best represented taxonomic groups (each with 27% of total articles), followed by fishes (17%), aquatic invertebrates (15%), birds (12%), herpetofauna (10%), and mammals (3%). Comparisons involving two or more species (65%) were more common than intraspecific surveys (35%). Interestingly, 45% of studies included comparisons across habitat gradients, and 35% of the 40 articles assessed patterns of gene flow. In terms of the main approaches used to generate and analyze data (**Figure 2**), a large proportion of studies used information from molecular phylogenetics (63%), phylogeography (50%) and morphology (42%). To our surprise, only few surveys of population genetic structure were conducted in the topics (27% of the total), with a handful of those also incorporating information from genome scans of selection (10%) or from landscape genetics (7.5%). As expected for any integrative field of research, ecological speciation was usually assessed by combining two or more approaches (80% of articles). Most combinations (83%) included phylogenetic or phylogeographic datasets, exemplifying the reliance of the field on molecular genealogical information.

What are the reasons for the rarity of ecological speciation research in tropical regions? First of all, basic taxonomic, ecological and distributional data are usually either inadequate or simply non-existent for most tropical biotas. Such information is needed to identify ecological ‘opportunities’ for speciation but is currently limited and hard to collect in the tropics compared to temperate regions because of the tropics’ inherently high levels of biodiversity, endemism and remoteness. In addition, tropical ecosystems are typically found in developing countries, where essential resources (e.g., research funding, *in situ* capacity and accessible cataloged bio inventories) are the scarcest. The latter is consistent with the positive correlation found in another review (Beheregaray, 2008) between global research productivity of population-level genealogical surveys with country’s wealth.

The low number of studies on ecological speciation might also be related to a historic research bias toward clarifying speciation timing and rates of diversification in the tropics. Many studies of speciation have used molecular phylogenies (e.g., **Figure 2**) to clarify temporal and spatial patterns of diversification in tropical ecosystems. These surveys and meta-analyses have invariably compared lineages at or above the species level and framed pattern interpretation within historical biogeographic and macroevolutionary contexts. They have contributed substantially to our understanding of tropical diversity – such as improving the quality of the contentious debate about Tertiary versus Quaternary speciation timing (Pennington et al., 2004; Rull, 2008; Hoorn et al., 2010b, 2011; Antonelli and Sanmartín, 2011) and clarifying general patterns of diversity and diversification (e.g., for birds, Salisbury et al., 2012; plants, Richardson et al., 2001; Hughes et al., 2013; and mammals, Rolland et al., 2014). However, we believe they have also created a paradigm about tropical speciation that often ignores population-level information and the roles of ecology and natural selection in the origin of species. Any biogeographic scenario, recent or historical, begins with population differentiation, a process underpinned by changes in the physical and biotic environments of populations (Darwin, 1859; Avise, 2000). Ecological considerations at the scale of demes are therefore relevant to understanding not only underpinnings of population divergence and incipient speciation but also to inform large-scale biogeographical patterns (Wiens and Donoghue, 2004). Indeed, there is mounting evidence from multiple taxa and ecosystems suggesting that divergent natural selection might be an important driver of biodiversity (Schluter, 2000; Coyne and Orr, 2004; Rundle and Nosil, 2005; Schluter and Conte, 2009), including the tropics (Smith et al., 1997, 2001; Schneider et al., 1999; Garcia-Paris et al., 2000; Ogden and Thorpe, 2002; Lopez-Fernandez et al., 2010; Cooke et al., 2012a,b,c,d, 2014).

## AN INTEGRATED GENETIC-BASED FRAMEWORK FOR STUDYING ECOLOGICAL SPECIATION IN THE TROPICS

**FIGURE 3:**
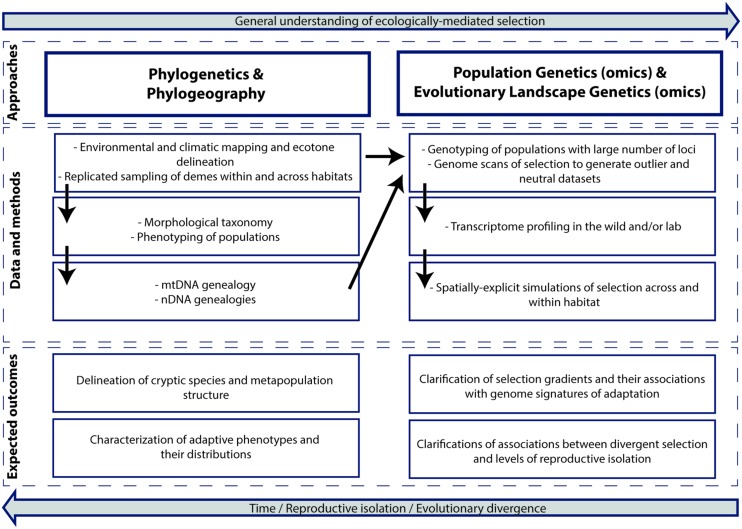
**A conceptual and analytical framework to study ecological speciation in the tropics based on genetic, genomic and non-genetic data and simulation modeling (see text for details).** The upper boxes show approaches that when integrated can improve understanding of ecologically mediated selection. The middle boxes show the main methods and types of data, whereas the bottom boxes show expected outcomes. The diagram exemplifies a single-taxon study but it can also be extended to comparative surveys. This framework can be applied to other ecosystems.

We now describe a framework to study ecological speciation in the tropics that integrates genomic (or genetic) data from populations with simulation modeling specific for ELG (summarized in **Box I**; **Figure 3**). Although the primary impetus is to assess the role of ecology as a driver of biodiversity, this type of research also generates valuable information for several data-deficient areas in tropical research. These include improvements of biological inventory databases and taxonomy, the discovery and delineation of cryptic species, selection gradients, ecotones and regional hotspots for conservation management, and the clarification of speciation timing, patterns of connectivity and metapopulation structure.

**BOX I. Rationale.**

The emphasis of the framework is about assessing gene flow among populations (or preferably, among adaptive phenotypes) distributed across heterogeneous environments or ecotones while controlling for spatial genetic autocorrelation and vicariant history. In this scenario, evidence for ecological speciation is obtained if a positive correlation is found between genetic differentiation and environmental (or phenotypic) divergence, after the effects of geographic distance (Shafer and Wolf, 2013; Sexton et al., 2014) and historical biogeography between populations are accounted for. This is considered evidence because ecologically divergent selection across environments can reduce gene flow between populations and in this way drive local adaptation and population divergence (Schluter, 2000; Räsänen and Hendry, 2008; Nosil, 2012). Notwithstanding the focus on population genetics, the framework also integrates genealogical information from phylogenetics and phylogeography to disentangle ecological divergence from vicariant biogeographic history. This distinction is needed because ecological speciation usually requires a study system in which the existence of an allopatric phase is very unlikely in the context of evolutionary history (Endler, 1982; Coyne, 2007; Nosil, 2008). In an additional conceptual and analytical step, we synergistically combine the outcomes of above-mentioned empirical analyses of gene flow and population diversification with spatially explicit simulations in evolutionary landscape genetics, ELG (Landguth et al., 2012, 2014). These ELG simulations can be used to statistically assess inferred scenarios of environmentally induced population divergence. They can also shed light on the role of landscape structure and individual based organism response to landscape structure in the evolution of reproductive isolation (**Box II**). Although this framework was conceived to assess ecological speciation in the tropics, it can be applied to other ecosystems and to most sexually reproducing species. This is particularly true for biotas in temperate regions for which *a priori* information about traits subject to divergent selection is more abundant and for which better logistics allow greater experimental tractability (Nosil, 2012) compared to biotas from tropical regions.

Below we introduce the main components of the framework and discuss design and experimental feasibility within the context of tropical research. We also provide a more detailed description of simulations and ELG (two largely unexplored approaches in ecological speciation) and their expected contributions. This is followed by a performance assessment of the proposed research framework based on published studies of population diversification and ecological speciation of Amazonian fishes.

### SAMPLING STRATEGIES AND PHENOTYPIC INFORMATION

Beginning with sampling a particular study system, the framework allows for both single-taxon studies and for comparative analyses that use population samples from codistributed species. Although population-level sampling and data collection for codistributed species can be logistically intricate and expensive, comparative studies are often more powerful and rewarding than single taxon surveys. Such studies allow for the identification of biogeographic histories that are shared among biotas (Avise, 1992; Bernatchez and Wilson, 1998) and regions that promote rapid adaptive evolution, have high concentrations of historically isolated populations, or both (Davis et al., 2008; Carnaval et al., 2009). In this case, sampling species across a range of mobility and life histories provides a powerful strategy to make generalizations about the effects of landscape history and environmental structure on gene flow (e.g., Galarza et al., 2009). Our proposal to integrate diverse empirical and simulated approaches (**Figure 3**) is valuable for comparative surveys because the applicability of individual approaches for assessing ecological speciation scenarios is expected to vary among groups of organisms (Nosil, 2012).

Natural selection works on phenotype, and the connections between selection on ordinary phenotypic traits and reproductive isolation are often strong and straightforward (Schluter, 2001). On the other hand, the complexity of both the environmental variation and the genetic basis of adaptive phenotypes hamper our ability to use genetic data to map adaptive variation over the landscape (Lowry, 2010; Schoville et al., 2012). The spatial delineation of ecologically relevant phenotypes (i.e., adaptive phenotypes; **Figure 3**) or of traits related to reproductive isolation can be fundamentally important to generate hypotheses about divergence across environmental gradients and to inform sampling strategies. In cases where *a priori* information is available about the distribution of phenotypically divergent ecotypes within species or between closely related lineages (e.g., Bernatchez et al., 2010) it might be prudent to invest in replicated population sampling across geographically separate but similar environments (Smith et al., 1997). Here, evidence for independently repeated genetic divergence (neutral or adaptive) in divergent phenotypes correlated with similar environmental gradients is strong evidence for selection (Bernatchez et al., 2010; Rosenblum and Harmon, 2011).

Regardless of whether a single or multiple species are being targeted, intensive sampling (both in number of individuals per deme and in number of demes) is required to ensure rigorous statistical analyses. Within lineages, sampling should be hierarchical to enable analyses of spatial dependence: the design should be spatially nested regarding dispersal potential to capture the degree of autocorrelated genetic data (due to daily dispersal) and the environmental-species relationships (due to beyond natal dispersal; Manel et al., 2010).

The rigorous sampling regime proposed here contrasts remarkably with the sparse sampling strategies generally used in phylogeographic and phylogenetic studies of tropical biotas (Beheregaray, 2008; Rull, 2008; Antonelli and Sanmartín, 2011). Although intensive population sampling might be seen prohibitive for some projects, efforts to document biodiversity in the tropics have increased and become more integrated in recent years (Janzen et al., 2009). Consistent with this view, responsible collection of specimens and associated data and openly sharing of this knowledge have been advocated (Rocha et al., 2014). Similarly, the field of biogeography seems to be experiencing a renaissance for inter-disciplinary research associated in part with the realized and potential impact of rapid data accumulation for poorly studied regions (Dawson et al., 2013). Altogether, these and other developments (e.g., the recently created open access journal *Scientific Data*) should contribute to improving and accelerating initiatives supporting bioinventory collections and population sampling in the tropics. Population genetic surveys should tag along to ensure that tissue samples for DNA analysis (and in some cases for transcriptomics, **Figure 3**), are collected together with voucher specimens and other key associated data such as phenotypic information.

### LANDSCAPE MAPPING

Landscape (or riverscape or seascape) mapping is an important step that should be carried out in parallel with sampling design. A landscape is an area that is heterogeneous with regard to at least one variable of interest (Turner et al., 2001). A landscape with low permeability can decrease gene flow, while increasing genetic drift and population structure. The purpose of incorporating such information is to identify environmental and climatic gradients that might be impacting on both the structural and functional habitats of study species. This should improve the delineation of ecotones or habitat transition areas and thus inform on the most appropriate sampling design. It also creates scenarios to anchor empirical analyses of gene flow and simulations in ELG (discussed below). Mapping can be done based on data collected in the field or using existing GIS datasets that contain summary statistics for variables of interest, such as climate, landcover, structural habitat, topography, disturbance, and ecosystem productivity. For example, climate data can be gathered from WorldClim (Schoville et al., 2012) and Microclim (Kearney et al., 2014). Looking ahead, the increase availability of rich environmental and climatic data is expected to enable sophisticated functional analyses that can potentially be used in ecological speciation research. For instance, mechanistic niche models or probability of occurrence maps that identify functional traits that limit distributions (e.g., physiological responses and constraints) provide a view of the fundamental niche that can then be mapped to the landscape (Kearney and Porter, 2009). These traits can be allowed to evolve (e.g., by incorporating selection and heritability) to explore the potential impact of evolution and to predict adaptive dynamics and distribution shifts (Kearney and Porter, 2009; Hoffmann and Sgro, 2011). Rich ecological datasets that include measures of resource availability and biotic interactions are also expected to positively impact ELG by providing an opportunity to test whether specific ecological factors drive adaptive genetic variation (Schoville et al., 2012).

### PHYLOGENETICS AND PHYLOGEOGRAPHY

The integration of molecular genealogical information from phylogenetic and phylogeographic analyses into the framework is a prerequisite for subsequent analyses of intraspecific (i.e., within lineages) gene flow. Two main reasons account for this requirement. First, these analyses enable the discovery and spatial delineation of historically isolated lineages and hidden biodiversity, such as evolutionarily significant units (ESUs) and cryptic species. Levels of species richness and morphologically cryptic biodiversity have been grossly underdocumented in assessments of tropical biodiversity (e.g., Parra-Olea and Wake, 2001; Cooke et al., 2012d; Funk et al., 2012) because these rarely employ thorough morphology-based analyses, intensive population sampling and DNA-based methods (Beheregaray and Caccone, 2007; Beheregaray, 2008). For instance, our comparative research program on the evolution of Amazonian fishes focused on species suspected to be represented by a single evolutionary lineage across the range of each taxon. We sampled populations from four nominal taxa (i.e., species) from a project on upper reaches of the Negro river and from five nominal taxa for the water color project described below. After performing genealogical and population genetic analyses it became evident that we were actually working with, respectively, a minimum of eight (e.g., Cooke et al., 2009; Sistrom et al., 2009; Piggott et al., 2011) and eleven (e.g., Cooke et al., 2012a,b,c,d, 2014) ancient cryptic species in each project. Notwithstanding the value of molecular data, we recognize that morphology-based taxonomy has a central and unrivaled position in biodiversity research (Schlick-Steiner et al., 2007) and advocate for resource investment toward traditional taxonomy and museum collections to ensure that newly reported cryptic species will be properly cataloged and described following discovery.

The second reason for integrating molecular genealogical studies is that they add an essential component to the understanding of patterns of population structure and levels of reproductive isolation: time. Temporal changes in the physical and biotic environment of a population lead to demographic variations that are correlated with the structure of population genealogies (Avise, 2000). Phylogeographic studies, when integrated with information from historical disciplines of Earth sciences, can potentially describe the chronology of demographic variation and reproductive isolation of population units (Avise et al., 1998; Hewitt, 2001). As such, they can disentangle scenarios of relatively recent divergence due to ecology – the setting targeted by studies of ecological speciation, from patterns of allopatric divergence due to vicariant biogeographic history. Phylogeographic analyses can also address questions about the species divergence process that cannot be addressed without genealogical data. These include clarifying how evolutionary divergence proceeds in the context of colonization and ecological opportunity (Beheregaray et al., 2004), and how repeated distributional shifts may have constrained diversification to taxa in which reproductive isolation apparently evolves very quickly (Carstens and Knowles, 2006).

### POPULATION STRUCTURE, GENE FLOW AND GENOME SCANS OF SELECTION

In this step, DNA markers are used to genotype individuals across heterogeneous environments with the aim of clarifying patterns of gene flow and identifying the spatial locations of genetic discontinuities (i.e., population boundaries). This is a key step in the framework that should be done at the metapopulation level after historical diversification has been accounted for (as above). The expectation is that during ecological speciation, ecological or environmental distance (analogous to geographic distance), reduces homogenizing gene flow and correlates to genetic population differentiation. These patterns are now considered common in nature and usually referred to as isolation-by-ecology (Shafer and Wolf, 2013), or isolation-by-environment (Sexton et al., 2014). Evidence indicates that adaptive divergence between selective environments constrains gene flow through selection against either immigrants or hybrids (Schluter, 2000; Räsänen and Hendry, 2008). Similar to what happens in allopatric and parapatric speciation, ecologically induced population differentiation might not necessarily produce complete reproductive isolation or new species (Hendry, 2009; Nosil, 2012).

Although isolation-by-ecology can be detected with relatively small neutral molecular datasets such as microsatellites and AFLPs (Shafer and Wolf, 2013; see below for examples), next-generation-sequencing (NGS) methods now offer the possibility of identifying and typing 1000s of genetic markers (i.e., SNPs) for population genomic analysis. These included genotyping-by-sequencing methods that enable screening SNPs throughout the genome of non-model species in a relatively inexpensive manner (Narum et al., 2013). Using a large number of genetic markers is important in ecological speciation research. These markers can clarify evolutionary relationships between populations, ecotypes and lineages, while improving delineation of barriers to gene flow, selection gradients and patterns of isolation-by-ecology caused by demographic factors. Simultaneously, these large datasets can be used to identify genomic regions (or specific loci) that show evidence of divergent selection (Luikart et al., 2003; Seehausen et al., 2014). In population genomics, the latter step is often done using genome scans that compare allelic variation at markers spread throughout the genome in many individuals from ecologically different populations or species. During genome scans of selection, markers that potentially carry the signature of natural selection (i.e., ‘outlier’ loci used to characterize population adaptations) will be distinguished from neutral markers (i.e., markers used to infer population parameters and phylogeography) because they exhibit exceptionally high levels of differentiation (Luikart et al., 2003; Beaumont, 2005). The premise of the method is that drift, inbreeding and gene flow usually have genome wide effects, whereas selection leaves signatures only at those loci that are adaptive to a particular scenario (Luikart et al., 2003; Seehausen et al., 2014). As such, differentiation will accumulate in regions under selection, whereas in other regions, genetic drift will require longer periods of time to accumulate (Wu, 2001; Nosil et al., 2009). By extension, sympatric or parapatric populations evolving in the presence of gene flow are predicted to show greater heterogeneity across the genome than spatially isolated populations (Wu, 2001; Nosil et al., 2009; but see Renaut et al., 2013).

### SIMULATION MODELING FOR EVOLUTIONARY LANDSCAPE GENETICS

In this section, we provide an introduction about simulations and landscape genetics (for a review see, Landguth et al., 2014) and list general questions that can be addressed with this type of approach (**Box II**). Simulations have provided many important findings in various disciplines (e.g., Grimm and Railsback, 2005), and are increasingly accepted by empiricists (Jeltsch et al., 2013). The quasi-experimental framework of simulation models offers several important benefits for scientific research. For example, the ability to repeat simulations of a system with varying parameters (sensitivity analysis) allows researchers to assess how much confidence we can put in the conclusions derived from simulations and to predict how a system or its behavior will change if certain processes are altered. With empirical data, the range of parameter values and assumptions that can be tested is usually much more limited, potentially leading to weaker or more uncertain inferences. Simulations can also mimic perfect sampling conditions, which can lead to stronger inferences (see review in Balkenhol and Fortin, 2014). In sum, simulation modeling can be used to predict and explain, guide sampling and data collection, illuminate core dynamics of a system, discover new questions, bound outcomes to plausible ranges, quantify uncertainties, and train students and practitioners (Epstein, 2007).

**BOX II — General aims (left) and questions that can be addressed with ELG simulations.**

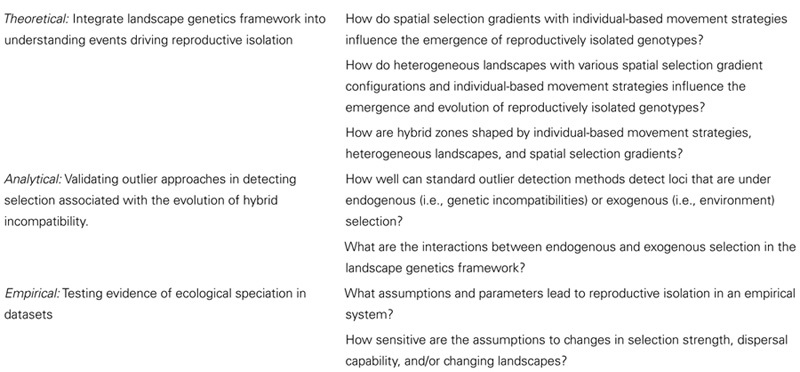

Simulations have been used in population and landscape genetics for many years, and the availability of software for simulating genetic data is increasing steadily (reviewed in Hoban et al., 2012; Hoban, 2014). However, there are important differences between ELG simulations and ‘classic’ population genetic simulations. First, many genetic simulation approaches generate genetic data (i.e., summary statistics) only at the population-level. In contrast, many ELG approaches produce genetic data for every individual (even if these are grouped into populations), and thus rely on individual-based models (IBMs). IBMs are classes of computational models for simulating the actions and interactions of autonomous individuals. Individuals can differ in their attributes (e.g., males vs. females) and these attributes influence their actions (e.g., different dispersal for males vs. females), as well as their reactions toward each other (e.g., mating strategies) or to other simulation settings (e.g., varying propensity to cross simulated barriers for males vs. females).

A second major characteristic of ELG simulations is the fact they are always based on spatially explicit models. These models are defined by placing individuals or groups of individuals (i.e., populations) on 1- or 2-dimensional regular lattices, or in irregular (x-, y-) coordinate space. Specific rules in the model then define how individuals move and interact across space, for example by defining the distances they can move away from their birth location, or the distance within which they can find a mating partner. Population genetic simulations are often also spatially explicit (e.g., Balloux, 2001), but while simulating population genetic data without space is possible, this is not the case for ELG simulations. In addition to space, another vital feature of ELG simulations is the direct incorporation of environmental heterogeneity into the underlying model. This usually requires a spatial representation of the environment that individuals are placed in. Importantly, this environment is spatially variable (i.e., non-homogeneous) in space, and potentially also in time, and it directly affects some or all of the essential processes included in the model. Thus, the rules that govern the actions and reactions of simulated individuals not only depend on pure space, but also on the user-defined environmental heterogeneity included in the model. This is the key distinguishing feature between population genetic and landscape genetic simulation modeling.

The ELG simulation modeling implemented in our ecological speciation framework includes an additional component: it specifically integrates landscape genetics and evolutionary genetics, focusing on how space and selection impacts on the evolution of reproductive isolation (speciation). For example, selection can be controlled via fitness landscape surfaces (Wright, 1932; Gavrilets, 2004) that determine the genotype-dependent viability of offspring in a spatially explicit setting. Let us consider a single bi-allelic locus model in which three relative fitness surfaces are specified for the three genotypes (AA, Aa, and aa). Selection is then implemented through differential survival of offspring as a function of the relative fitness of its genotype [e.g., determined in ‘water color’ in our Amazonian study (Cooke et al., 2014); see below] at the location on that surface where the dispersing individual settles. Then, through ELG simulations (e.g., Landguth et al., 2012), one can explore modeling of natural selection in landscape genetics with individual organism dynamics. Through sensitivity and uncertainty analysis, a factorial study design can be used with a range of species-specific dispersal strategies (controlling for gene flow) across landscape resistance scenarios. In addition, a range of genetic parameters and exogenous selection (arising from the environment) as well as endogenous selection (arising from genetic incompatibilities) can be explored while tracking the evolution of reproductive isolation (e.g., Cooke et al., 2014). Note that, when attempting to understand endogenous selection in spatial settings, fitness is determined by epistatic interactions, in form of the well-known Dobzhansky–Muller model (Dobzhansky, 1937; Muller, 1942; e.g., Eppstein et al., 2009). Thus, extensions to multi-loci selection models must be considered for addressing these questions.

## FRAMEWORK TESTING: COMPARATIVE ANALYSIS OF PHYLOGEOGRAPHY AND ECOLOGICAL SPECIATION IN AMAZONIAN FISHES

**FIGURE 4:**
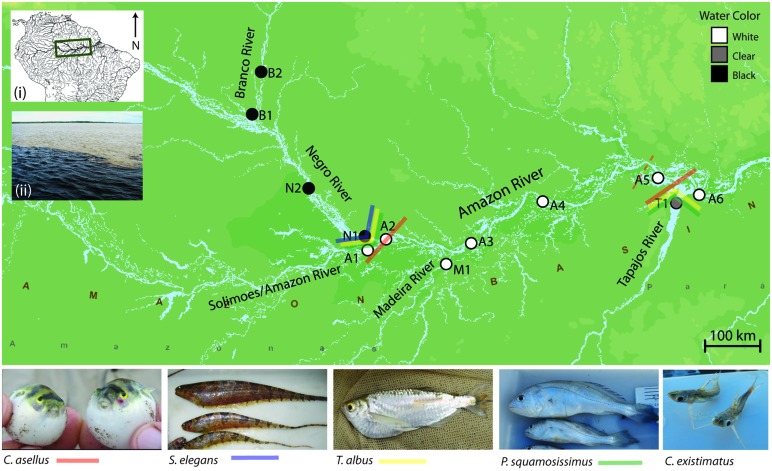
**Map showing sampling sites and major population boundaries identified for each of the five study species (excluding *C. existimatus*).** Analyses used either a combination, or all of the following analytical methods based on nDNA and mtDNA sequences and on AFLP markers: nested clade phylogeographic analysis (Templeton et al., 1987), STRUCTURE (Falush et al., 2003), mtDNA Φ_ST_ (Excoffier et al., 1992), and AFLP *F*_ST_ (Lynch and Milligan, 1994). Site abbreviations are: N, Negro River; B, Branco River; A, Amazon River; M, Madeira River; T, Tapajós River.

Here, we provide a number of examples from our published work (Cooke et al., 2012a,b,c,d, 2014) that illustrate how integrating population genetics, genome scans of selection, ELG and sequenced-based phylogeographic and phylogenetic methods can be used to assess the relative influence of environmental gradients and biogeographic history in shaping Amazonian fish biodiversity. The studies used a thorough sampling design and amassed 905 individuals from 48 putative populations of five taxonomically diverse and ecologically distinct fish species endemic to Amazonia (**Figure 4**). These included freshwater representatives of marine-derived lineages from two orders; a tetraodontiform (the puffer *Colomesus asellus*) and a perciform (the croaker *Plagioscion squamosissimus*) and ancient freshwater lineages representing three Gondwana-relict orders; a characiform (the characin *Triportheus albus*), a siluriform (the catfish *Centromochlus existimatus*) and a gymnotiform (the electric fish *Steatogenys elegans*). Nuclear and mitochondrial DNA (mtDNA) sequences were used for genealogical analyses and AFLP datasets were used for analyses of gene flow and genome scans. Readers interested about species-specific datasets, analytical methods, and detailed biogeographic reconstructions should refer to our five publications (Cooke et al., 2012a,b,c,d, 2014).

**FIGURE 5:**
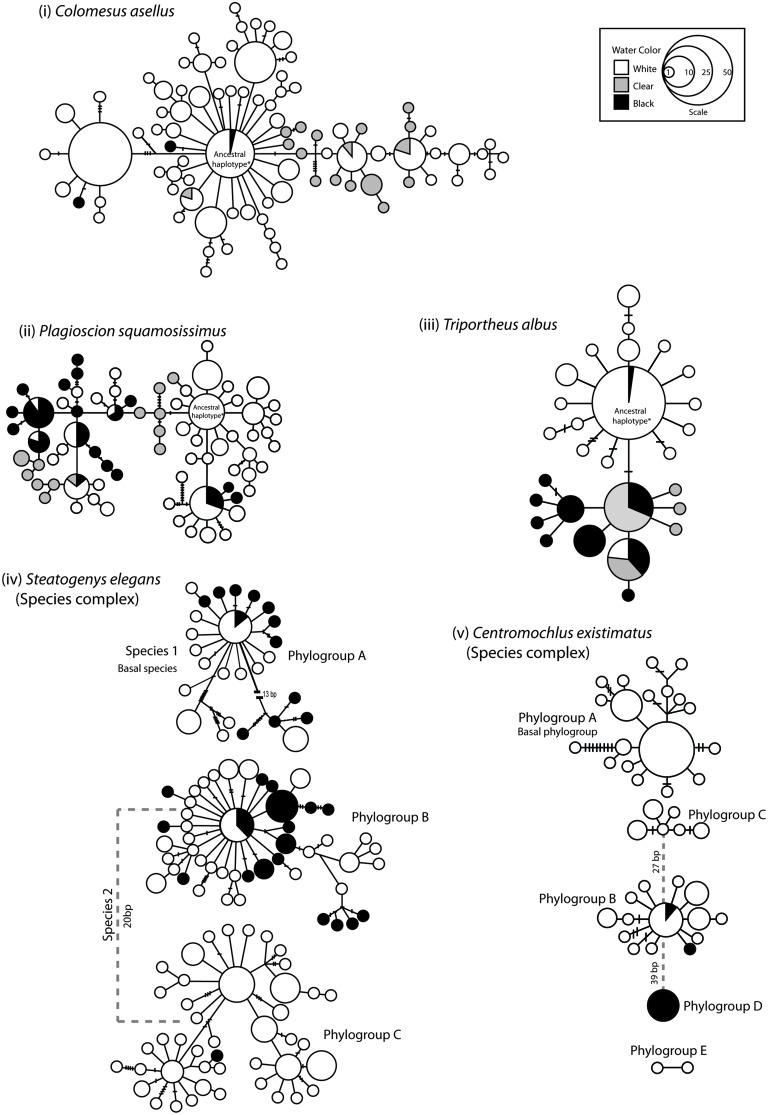
**Genealogical relationships for each species and species complex based on mtDNA ATPase 6 and 8 sequences.** Relationships among haplotypes were estimated following Templeton et al. (1992). Each circle denotes a unique haplotype and the area of the circle is proportional to its frequency. Lines joining haplotypes represent one mutation and small lines are missing haplotypes (not sampled or extinct). The shade/s of the circle represents the water color of the sampling locality. ‘*Ancestral haplotype’ denotes the haplotypes considered as ancestral based on statistical parsimony.

**FIGURE 6:**
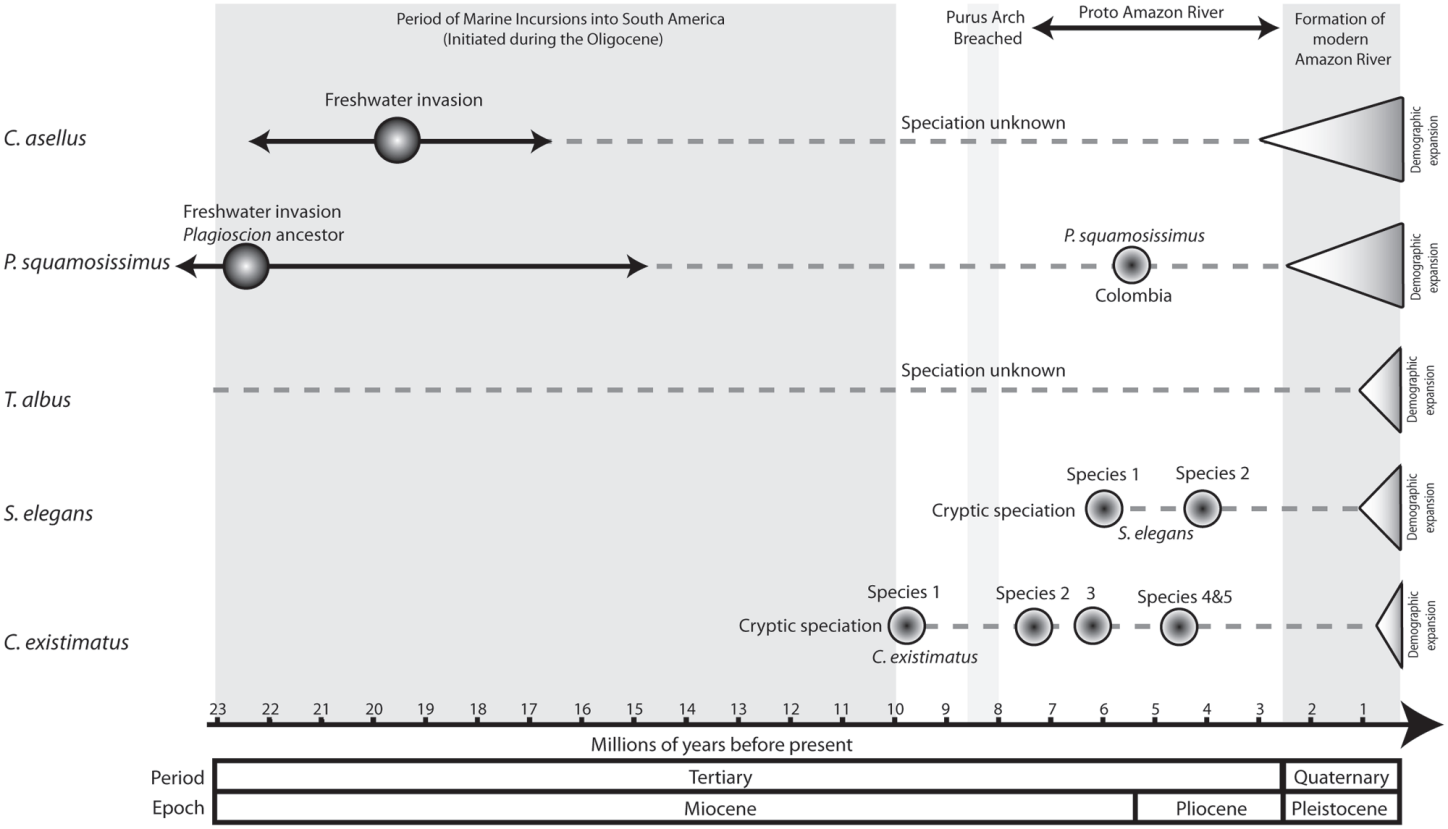
**Comparative chronogram of key speciation and demographic events of the five study species.** For *C. asellus* the estimated date of freshwater invasion is based on fossil data after Monsch (1998). Estimated dates of marine incursions, the breach of the Purus Arch and subsequent formation of the Proto Amazon River are after Lundberg et al. (1998). The date estimate for the final formation of the modern Amazon River is after Campbell et al. (2006) following the erosion of the Madre de Dios formation. Date estimates of demographic expansions are based on mismatch analysis (**Table 1**), and speciation events are based on molecular dating from phylogenetic analysis using BEAST 1.6.1 (Drummond and Rambaut, 2007). Where phylogenetic methods were not employed, the timing of speciation has been stated as unknown.

The five species are largely codistributed, yet results indicate they have very different evolutionary histories. Phylogenetic analyses unexpectedly revealed five cryptic species within the catfish *C. existimatus* and two fully sympatric cryptic species within the electric fish *S. elegans* (**Figure 5**). Molecular dating indicates that these lineages all diverged during middle to late Miocene (**Figure 6**). In contrast, there was no evidence for cryptic species within the marine-derived taxa or within *T. albus* (**Figures 5** and **6**). Despite these differences, however, several congruous patterns that may actually reflect general forces shaping freshwater biodiversity in Amazonia were detected. Below we introduce the study region and summarize key comparative findings about inferred spatial arrangements of neutral and putatively adaptive genetic diversity. These are discussed within the contexts of geomorphological history of Amazonia and divergent selection.

### STUDY SYSTEM

The Amazonian aquatic environment sustains dramatic hydrochemical and ecological gradients that impose physiological constraints upon its aquatic communities (Junk et al., 1983; Henderson and Crampton, 1997; Rodriguez and Lewis, 1997; Saint-Paul et al., 2000; Petry et al., 2003). These aquatic conditions have been grouped into three water types or ‘colors’ and are differentiated largely by sediment composition, geochemistry and optical characteristics (Sioli, 1984): (i) white water, which has an Andean origin, is turbid in nature and is characterized by large amounts of dissolved solids and a neutral pH; (ii) clear water, which is comparatively transparent and contains low content of dissolved solids and a neutral pH; and (iii) black water, which is transparent yet stained by tannins and humic acids leached from vegetation and has low pH (pH ~5 or lower). Both clear and black water differ from white water in that they are craton born, draining the Brazilian and Guyana shields, respectively (Hoorn et al., 2010a). As a result, their sediment composition and channel formation are different from the fast-turbid Andean white waters. Major ecological gradients have been shown to generate biodiversity via divergent natural selection elsewhere (Endler, 1973; Smith et al., 1997, 2001). This led to the prediction that major differences in water color between rivers of the Amazon Basin provide ecological opportunities for natural selection to drive genetic divergence between populations of aquatic organisms.

The study system within the Amazon Basin consists of the five major rivers representing white, black and clear waters: the Amazon (white), Madeira (white), Branco (seasonally black), Negro (black), and Tapajós (clear; **Figure 4**). The study area encompasses two putatively strong selection gradients or ecotones: where the black waters of the Negro River meets the white waters of the Amazon River, and where the clear waters of the Tapajós River meets the white waters of the Amazon River. Additionally, the transect allows testing for genetic structure geographically associated with river confluence. That is because two controls in which rivers of the same water color meet were included: the confluence of the black waters of the Branco and Negro Rivers and the confluence of the white waters of the Madeira and Amazon Rivers (**Figure 4**).

### COMPARATIVE PATTERNS

Despite differences in ecology and phylogenetic history, strong evidence indicates that the biogeographic and ecological contexts of the Amazon Basin have promoted largely congruent fine-scale phylogeographic and population-level structuring. From a genealogical perspective, all species showed strong signals of demographic expansion and in every case the timing of these occurred well within the Quaternary period, with the exception of *C. asellus* whose expansion began ~0.5 Ma earlier (**Table 1**).

**Table 1:** Historical demographic analyses based on DNA sequences for all species and species complexes.

Species (Phylogroup)	SSD	*P*	R	*P*	F_S_	*P*	Time since expansion (Ma)	Range (α = 0.05; Ma)
*C. asellus*	0.007	0.54	0.012	0.681	-24.90	<0.001	~3	1 – 4.3
*P. squamosissimus*	0.0046	0.619	0.0072	0.913	-25.19	<0.001	~2.6	1 – 3.6
*T. albus*	0.0066	0.45	0.0267	0.736	-6.16	0.062	~1	0.2 – 2
*S. elegans* (Phylogroup B)	0.001	0.325	0.025	0.319	-26.00	<0.001	~1	0.9 – 1.6
*S. elegans* (Phylogroup C)	0.002	0.804	0.003	0.997	-25.05	<0.001	~1	0.2 – 6
*C. existimatus* (Phylogroup A)	0.012	0.243	0.017	0.994	-12.74	<0.001	~0.5	0.02 – 1
*C. existimatus* (Phylogroup B)	0.005	0.394	0.059	0.354	-8.47	<0.001	~0.7	0.04 – 1

*Summaries include the Sum of squared deviations (SSD), Raggedness Index (R), and Fu’s test of neutrality (F_S_). Estimates of time since expansion are shown for analyses exhibiting evidence of demographic expansion. Phylogroups correspond with haplotype networks in **Figure 2**.*

From a population genetic perspective, all species and species complexes showed a predominant barrier to gene flow at the confluence of the Negro and the Amazon Rivers, and/or again at the confluence of the Tapajós and Amazon Rivers (**Figures 4 and 5**). No significant barrier to gene flow was identified at the confluence of the Madeira and Amazon Rivers in any species. Indeed, based on both mitochondrial and nuclear datasets, population structure was strongly associated with ‘water color’ but not with river system. For instance, statistically significant differentiation was consistently found between populations from rivers of different colors, but not between those from rivers of the same color (**Tables 2** and **3**; **Figure 4**). For *C. existimatus*, the large number of inferred cryptic species resulted in insufficient data for intraspecific analysis. However, the *C. existimatus* cryptic species ‘D’ endemic to black waters of the Negro River, appeared recently derived and genetically distinct from white water lineages (**Figure 5v**).

**Table 2:** Analysis of molecular variance (AMOVA) based on mtDNA sequences for three study species.

	Black vs. white water or white vs. clear water				White vs. white water			
Species	Source of Variation	% Variation	FI	*P*	Source of Variation	% Variation	FI	*P*
*C. asellus*	Between water colors	33.50	Φ_CT_0.3350	0.000*	Between white water	0.00	Φ_CT_:-0.014	0.398
	Between populations	3.29	Φ_SC_:0.0494	0.002*	Between populations	15.36	Φ_SC_:0.1514	0.000*
	Between individuals	63.21	Φ_ST_:0.3679	0.000*	Between individuals	86.09	Φ_ST_:0.1391	0.000*
*P. squamosissimus*	Between water colors	25.07	Φ_CT_:0.251	0.000*	Between white water	-2.91	Φ_CT_:-0.029	0.265
	Between populations	3.72	Φ_SC_:0.050	0.010*	Between populations	6.04	Φ_SC_:0.059	0.012*
	Between individuals	71.21	Φ_ST_:0.288	0.000*	Between individuals	96.87	Φ_ST_:0.031	0.015*
*T. albus*	Between water colors	46.29	Φ_CT_:0.463	0.008*	Between white water	-27.16	Φ_CT_:-0.272	0.665
	Between populations	13.50	Φ_SC_:0.251	0.000*	Between populations	45.09	Φ_SC_:0.355	0.000*
	Between individuals	40.21	Φ_ST_:0.598	0.000*	Between individuals	82.08	Φ_ST_:0.179	0.000*

Comparisons ‘between water colors’ are based on populations grouped according to river color. FI stands for Fixation Index, significant results are indicated by*.

**Table 3:** Analysis of molecular variance (AMOVA) based on AFLP data for three species and species complex.

	Black vs. white water or white vs. clear water				White vs. white water			

**Species**	**Source of Variation**	**% Variation**	**FI**	***P***	**Source of Variation**	**% Variation**	**FI**	***P***
*C. asellus*	Between water colors	3.00	Φ_RT_:0.030	0.000*	Between white water	0.00	Φ_RT_:0.4	0.235
	Between populations	7.00	Φ_PR_:0.067	0.000*	Between populations	8.00	Φ_PR_:0.081	0.000*
	Between individuals	91.00	Φ_PT_:0.095	0.000*	Between individuals	92.00	Φ_PT_:0.081	0.000*
*T. albus*	Between water colors	3	Φ_RT_:0.027	0.000*	Between white water	1	Φ_RT_:0.014	0.057
	Between populations	5	Φ_PR_:0.053	0.000*	Between populations	4	Φ_PR_:0.045	0.000*
	Between individuals	92	Φ_PT_:0.079	0.000*	Between individuals	94	Φ_PT_:0.058	0.000*
*S. elegans*	Between water colors	6	Φ_RT_:0.060	0.001*	Between white water	2	Φ_RT_:0.017	0.298
(Species 1)	Between populations	1	Φ_PR_:0.009	0.272	Between populations	4	Φ_PR_:0.042	0.174
	Between individuals	93	Φ_PT_:0.069	0.002*	Between individuals	94	Φ_PT_:0.058	0.024
*S. elegans*	Between water colors	2	Φ_RT_:0.020	0.050*	Between white water	0	Φ_RT_:-0.003	0.494
(Species 2)	Between populations	1	Φ_PR_:0.010	0.090	Between populations	1	Φ_PR_:0.011	0.145
	Between individuals	97	Φ_PT_:0.030	0.020*	Between individuals	99	Φ_PT_:0.008	0.192

Comparisons ‘between water colors’ are based on populations grouped according to river color. FI stands for Fixation Index, significant results are indicated by *.

**FIGURE 7:**
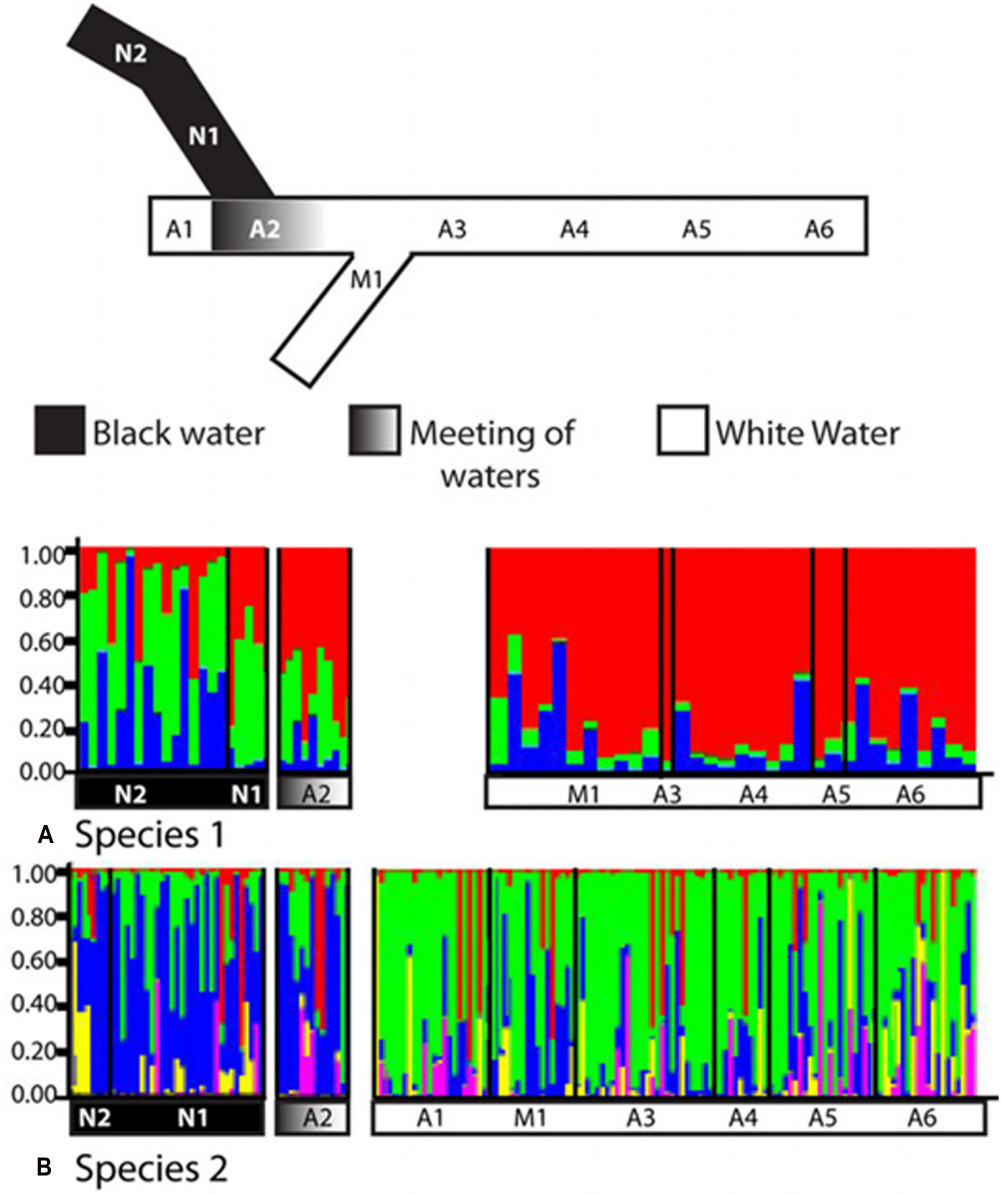
**Replicated ecological divergence in Amazonia due to water color in sympatric cryptic species of the electric fish *S. elegans*.** The diagram shows STRUCTURE results for **(A)** cryptic species 1 (*n* = 62) and for **(B)** cryptic species 2 (*n* = 171). Individuals are grouped by sampling location, and each individual is represented by one vertical column. Sample sites and water colors as listed on STRUCTURE graphs correspond to the sampling locations as shown in the simplified map above the graphs. Site abbreviations are: N, Negro River; A, Amazon River, M; Madeira River. Reproduced from Cooke et al. (2014).

Remarkably, ecologically driven divergence was also depicted in replicate over the riverscape in the two sympatric cryptic species discovered within the electric fish *S. elegans*. Here, intraspecific divergence within both sp. 1 and sp. 2 is due to a barrier to gene flow between black and white water, whereas no barrier to gene flow was identified between white water rivers (**Figure 7**; **Table 2**). These lineages likely exemplify “natural replicates of the ecological speciation in progress” (*sensu* Rosenblum and Harmon, 2011) and as such should assist with the discovery of general rules about divergent natural selection that may result in ecological speciation (Cooke et al., 2014).

Using outlier loci approaches based on genome scans, divergent selection was quantified between populations of *C. asellus*, *T. albus* and *S. elegans*. Heightened selection was detected at the interface of different water colors in the three species (**Table 4**), irrespective of river system and tributary arrangement.

**Table 4:** The average number of repeat outlier loci for pairwise comparisons between sites for three study species.

Species	Black vs. White	White vs. White	White vs. Clear
*Colomesus asellus*	NA	3.6	5.5
*Triportheus albus*	14	1.6	3
*Steatogenys elegans* (sp. 2)	4	1.3	NA

*Repeat outliers are those detected only in multiple comparisons, which corrects for type I errors. The ALFP data are based on 460 polymorphic loci for *C. asellus*, 360 polymorphic loci for *T. albus* and 310 polymorphic loci for *S. elegans*. NA, samples not available.*

### SIMULATING ELG ALONG THE AMAZON RIVERSCAPE

**FIGURE 8:**
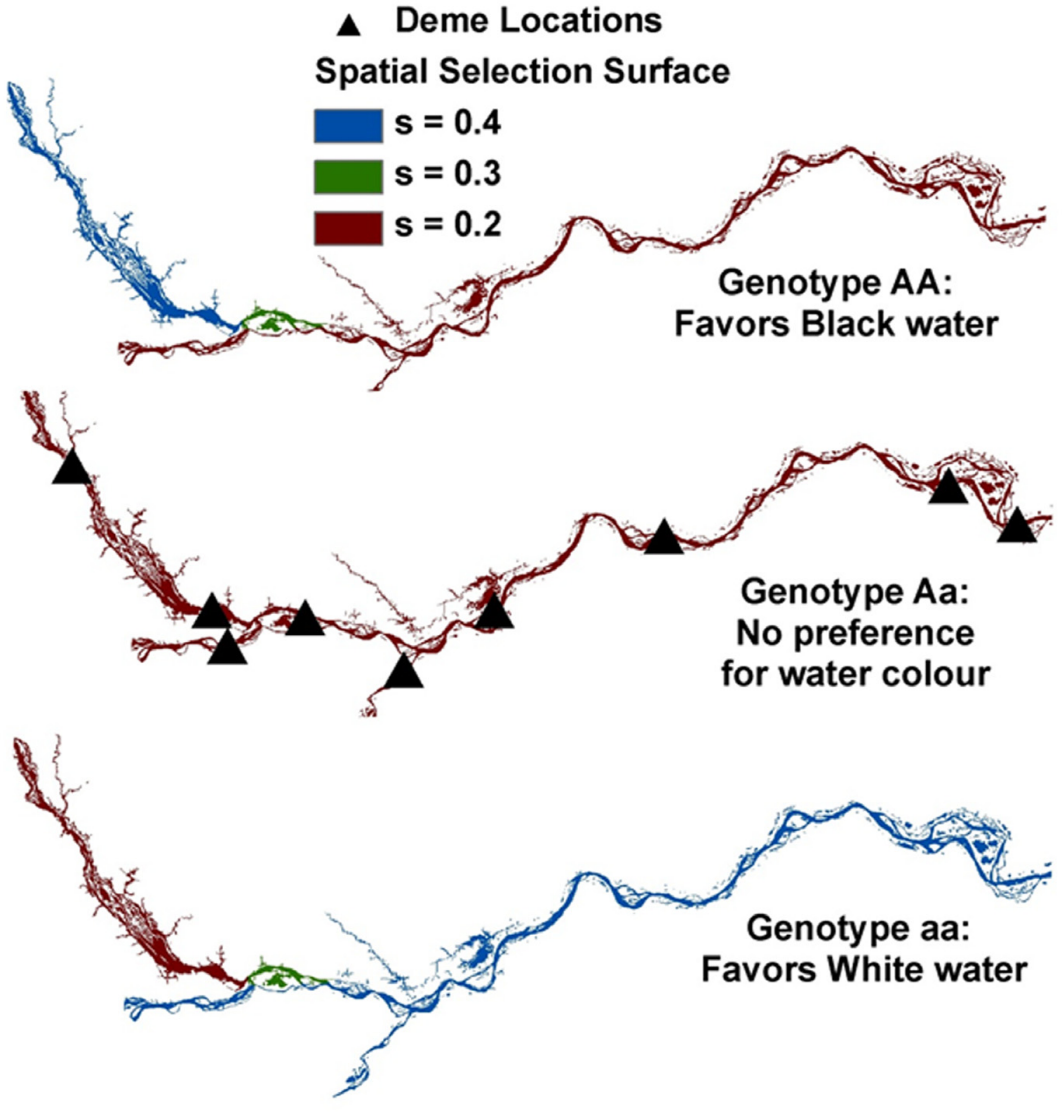
**Simulated ELG (evolutionary landscape genetics) along the Amazon Basin riverscape depicting our nine sampled demes or sites (triangles) in the Negro, Amazonas and Madeira Rivers.** For AA (top panel), relative fitness coefficients were used to favor black water (e.g., 0.4, 0.3, and 0.2 for black, mixed, and white waters, respectively). For aa (bottom panel), an opposite spatial selection gradient was implemented to favor white water (relative fitness coefficients of 0.2, 0.3, and 0.4 for black, mixed, and white waters, respectively). Aa (middle panel) received a uniform selection gradient (e.g., 0.2). Details about rivers are given in **Figure 4**.

Here we describe an ELG approach that incorporates selection to simulate genetic exchange along the Amazonian riverscape. Although this was used to explore the reasons behind the black water/white water population boundary detected in the electric fish *S. elegans* (**Figure 8**; Cooke et al., 2014), such approach can be readily extended to a wide range of studies of ecological speciation.

Simulations were conducted in the individual-based landscape genetics program CDPOP v1.2 (Landguth and Cushman, 2010; Landguth et al., 2012). Individual genetic exchange was simulated for over 100 non-overlapping generations as a function of individual-based movement, mating, dispersal, and selection, using 100 individuals spatially located at each of the nine populations in the study system. One locus was set to be under spatial selection and tied to ‘water color,’ and 19 loci were selectively neutral across a riverine distance surface that controlled for individual movement (mating and dispersal). Following similar studies (e.g., Landguth and Balkenhol, 2012) and expanding to a spatially explicit environmental gradient in a riverscape setting (see **Figure 8**), selection pressures were altered due to ‘water color’ between populations by considering three spatially explicit relative fitness surface scenarios. (i) No spatial selection gradient (‘uniform’): in this scenario, the three genotypes (AA, Aa, and aa) were being selected against, but uniformly across the ‘water color’ riverscape scenario, thus having no spatial dependency. (ii) Gentle spatial selection gradient (‘gentle’): here, a ‘gentle’ spatial selection gradient corresponding to the three river color locations and three genotypes was used. (iii) Steep spatial selection gradient (‘steep’): for this scenario, stronger spatial selection gradients were assigned to each genotype for black, mixed, and white waters. For further details, see Cooke et al. (2014).

In the *S. elegans* replicated system, population genetic analyses and *F*_ST_-based genome scans showed that recent divergence appeared linked to a major hydrochemical gradient within each cryptic species (**Tables 3** and **4**; **Figure 8**). Results from ELG simulations further corroborate these findings. These ELG simulations show that neutral data can give a low population differentiation signal (similar to the empirical neutral data findings based on AFLPs). They also show that selection-driven loci can respond with high population differentiation to the water color ecotone (similar to the empirical outlier loci findings).

The results link selection across an ecological gradient with reproductive isolation and it was speculated that assortative mating based on chemically different water types may be driving the divergence (Cooke et al., 2014). The rationale is that the major differences in pH and conductivity between waters of the Negro and Amazonas influence the transmission of electric signals used for courtship signaling and for precise synchronization of external fertilization – a hypothesis consistent with the idea that electric discharges in African electric fish are drivers of sympatric speciation (Feulner et al., 2006).

### EVOLUTIONARY PROCESSES SHAPPING AMAZONIAN FISH DIVERSITY: GEOMORPHOLOGICAL HISTORY AND DIVERGENT NATURAL SELECTION

The palaeogeographic and paleoenvironmental changes in South America during the Miocene are known to have profoundly affected the evolution of the Amazonian fish fauna. These changes include the uplift of the Andes and associated neotectonic events, the incursion of marine waters into previously freshwater systems and the dramatic reorientation of major river drainages (e.g., the Amazon River), (Lundberg et al., 1998; Lovejoy and Collette, 2001; Montoya-Burgos, 2003; Hubert and Renno, 2006; Lovejoy et al., 2006; Ruzzante et al., 2006; Hubert et al., 2007; Sistrom et al., 2009; Hoorn et al., 2010a,b; Cooke et al., 2012b,c,d). Indeed, phylogenetic analyses combined with fossil data indicate that the early Miocene marine incursions were likely responsible for the colonization and adaptation to freshwaters of the two marine-derived lineages we studied (**Figure 6**). Moreover, dating of speciation events within *Plagioscion*, *S. elegans* and *C. existimatus* are consistent with the Miocene diversification of fishes observed in the fossil record, as well as in other molecular studies (Rull, 2008; Sistrom et al., 2009; Piggott et al., 2011). Moving further in time, our analyses detected phylogeographic signals supporting easterly trajectories of colonization down the Amazon River and consistently strong demographic expansions for all species dated for the Quaternary (**Figures 5** and **6**; **Table 1**). These studies also suggest that the ancestral ecotype for each species was in Andean-derived white water (Cooke et al., 2012b,c,d, 2014). These events of population history are likely correlated with the final establishment of the modern Amazon River during late Pliocene-early Pleistocene (Rossetti et al., 2005; Campbell et al., 2006). This association was attributed to the availability of novel aquatic habitats along the vast Amazon and its catchment, which enabled range expansions and left concordant records in the population genealogies of codistributed species (**Figure 6**).

Although palaeogeographic events have clearly contributed to Amazonian fish diversity, the comparative analysis indicates that the force of divergent natural selection, in this case between water colors, deserves equal consideration. One of the primary tenets of ecological speciation theory is that high ecological opportunity, such as the colonization of new environments in the absence of predation and/or competition, will promote rapid population divergence resulting in speciation (Mayr, 1963; Schluter, 2000). Indeed, adaptive radiations following the colonization of new habitats are well documented in fish (Bernatchez and Wilson, 1998; Beheregaray et al., 2002). The simultaneous Quaternary expansion events of the five species into the Amazon River system may have presented the high ecological opportunity necessary for divergent natural selection and adaptation between Andean (white) and Craton-derived (black and clear) waters.

Classic signatures of vicariant biogeographic history such as genetic drift, inbreeding and migration have genome wide effects, while selection usually leaves signatures only at those loci that are adaptive or tightly linked to adaptive loci via hitchhiking (Luikart et al., 2003; Jensen et al., 2007). For this reason, *F*_ST_-based genome scans were employed to assess the role of selection in the origin and maintenance of population divergence between water colors. However, key to ecologically based divergent natural selection is that the process should be greater between selective environments than within (Schluter and Conte, 2009). Indeed, in each species, more outlier loci on average were found in pairwise comparisons between different water colors than in comparisons between the same colors (**Table 4**).

For *C. asellus*, divergent selection detected between the Tapajós and Amazon Rivers was a powerful yet recent phenomenon, such that genetic drift had not yet accumulated in selectively neutral genomic regions (Cooke et al., 2012b). In contrast, the effects of isolation by environment was suggested for *T. albus* between black, white and clear waters (Cooke et al., 2012a). For that species, a population boundary between water colors has also arisen within the selectively neutral AFLP loci and is particularly marked in the mitochondrial data (**Figure 5iii**). For *S. elegans*, both the empirical landscape genetics and simulated ELG explicitly linked selection-driven population genetic structure to the water color ecotone, validating results of *F*_ST_-based genome scans. By incorporating information about population history and by independently validating outlier results with simulations, we improved our ability to exclude type 1 errors normally associated with genome scans of selection (see Cooke et al., 2014 for details).

In the context of comparative biology, the use of genome scans revealed variable, yet valuable perspectives on the process of adaptive divergence across water colors, both within and between species. Although there is no certainty that ecologically diverged lineages will eventuate as reproductively isolated species (Futuyma, 1987; Via, 2009), the observation of isolation by environment within *T. albus* and speciation in the *S. elegans* complex across the same environmental gradient added credence to the assertion that water color is a general force shaping Amazonian freshwater biodiversity. Considering this, it may also be likely that divergent natural selection is the mechanism maintaining population boundaries observed between water colors for *P. squamosissimus* and *C. existimatus* (**Figures 5ii, v**).

The evidence for the role of divergent natural selection in these studies occupies a relatively recent time frame and appears associated with the formation of the modern Amazon River and its aquatic ecotones. Considering that time erases the signature of divergent selection within the genome, resulting in a more diffuse pattern of genetic divergence (Via, 2009), ecological speciation may not be just a recent phenomenon within the Amazon Basin. Here, a broad taxonomic range of species with different life histories were sampled and in every case there was evidence that water color influenced population divergence. Thus, the process of divergent natural selection is also likely to be widespread in the accumulation of contemporary diversity, which may shift the paradigm for future evolutionary research in Amazonia.

## FUTURE DIRECTIONS

Clarifying the role of ecology on population adaptation and divergence is crucial for understanding the origin of species and the evolutionary potential of biodiversity to respond to ongoing global change (Hoffmann and Sgro, 2011; Seehausen et al., 2014). Although the theory of adaptation is well developed, measuring the strength and characteristics of selection in nature remains a daunting task (Endler, 1986; Schluter, 1988; Jones et al., 2012). We generally lack knowledge about the genomic basis of population adaptation and divergence, especially for tropical organisms. Despite these gaps in knowledge, recent years have seen exciting developments due to the explosion of information for non-model organisms created by NGS technologies. While modest genetic datasets can certainly inform on ecological divergence that may result in ecological speciation (e.g., Cooke et al., 2012a,b, 2014), it is now feasible to ‘genomicize’ ecologically and evolutionarily important species at relatively low costs (Travers et al., 2007; Ellegren, 2008; Bernatchez et al., 2010; Seehausen et al., 2014). NGS resources relevant for ecological speciation research include genome-wide marker panels for population genomics, whole genome sequences and transcriptomes. These can be used to identify gene regions and traits involved in adaptation, to understand selective factors influencing adaptive variation and to assess evolutionary resilience.

As illustrated in this review, studies of ecological speciation in the tropics should merge analyses of genealogical history and gene flow with recent developments in spatial modeling and ELG simulations. This integration provides valuable biogeographic and environmental contexts to disclose associations between landscape features and evolutionary processes, including divergent natural selection. Such approaches, when combined with NGS datasets and with information about divergent phenotypes can be used to identify adaptive loci across the landscape (Lowry, 2010; Schoville et al., 2012), and to better assess the relative sensitivity of adaptive and neutral loci across environmental barriers and under a range of gene flow (Balkenhol and Landguth, 2011). However, researchers have to unambiguously determine whether markers are under selection and undoubtedly, this multigenic process encompasses additive, epistatic interaction, and pleiotropic effects. While several analytical challenges remain, ELG simulations in combination with powerful genomic datasets can provide a snapshot of the potential of evolutionary processes. Future research should address how landscape heterogeneity affects the generation of cluster of reproductively isolated genotypes through landscape restricted migration. Furthermore, how do spatial selection gradients influence the emergence of reproductively isolating clusters? These questions have direct relevance for mosaic hybrid zones (e.g., Ross and Harrison, 2002) and how they are shaped by individual-based movement strategies, heterogeneous landscapes, spatial selection gradients, and their interactions. Forthcoming ELG simulations should combine a range of landscape complexities (affecting dispersal) with spatial selection gradients and complex endogenous selection (i.e., multi-loci interactions, recombination, and mutational models) within the framework outlined here to understand processes controlling the emergence of reproductive isolation.

Ecological adaptation and divergence in the face of homogenizing gene flow is still a controversial topic. Many studies of ecological speciation in nature appear as correlative, are based on results from lab experiments (Nosil, 2012), and showed only weak associations between divergent selection and levels of reproductive isolation (Hendry, 2009). The latter is not unexpected because divergent selection acts on adaptive traits responsible for post- and prezygotic reproductive isolation along an evolutionary continuum that ranges from adaptive variation within panmictic populations to complete reproductive isolation between species (Schluter, 2001; Hendry, 2009). In fact, a recent meta-analysis suggests that genetic divergence induced by ecologically based divergent selection is pervasive across time-scales and taxa (Shafer and Wolf, 2013). Marine ecosystems are no exception, with two decades of phylogeographic research indicating that the combination of ecological divergence and partial isolation (parapatry) probably offers the richest opportunities for diversification in the sea (Bowen et al., 2013).

The time is right for implementing studies that synergistically generate and explore information from adaptive phenotypes, phylogeography, population genomics and simulations in ELG to understand ecological divergence and speciation in the tropics. Such endeavors are expected to challenge results from current surveys that assess tropical diversity based on sparse population sampling and on geographic models that do not incorporate selection. In doing so, they will complement historical biogeographic and evolutionary studies of diversification of tropical biotas. Integrative frameworks such as the one illustrated here have considerable potential to enhance conservation management in biodiversity rich ecosystems and to contribute toward a better understanding of how ecology, space and time interact with the genome.

## Conflict of Interest Statement

The authors declare that the research was conducted in the absence of any commercial or financial relationships that could be construed as a potential conflict of interest.
